# Backward Walking as a Rehabilitation Strategy in Parkinson’s Disease: A Focused Systematic Review

**DOI:** 10.3390/medicina62050867

**Published:** 2026-04-30

**Authors:** Monika Jadwiga Krefft, Paulina Magdalena Ostrowska, Rafał Studnicki, Rita Hansdorfer-Korzon

**Affiliations:** Department of Physiotherapy, Medical University of Gdansk, 7 Dębinki Street, 80-211 Gdańsk, Poland; monika.krefft@gumed.edu.pl (M.J.K.); rstudnicki@gumed.edu.pl (R.S.); rita.korzon@gumed.edu.pl (R.H.-K.)

**Keywords:** Parkinson disease, backward walking, rehabilitation strategy, fall risk, gait

## Abstract

*Background and Objectives*: Parkinson’s disease (PD) is a progressive neurodegenerative disorder in which gait and balance disturbances substantially increase the risk of falls and loss of independence. Pharmacological treatment alleviates several motor symptoms but has limited effects on postural instability. Backward walking (BW), a demanding locomotor task, has recently been investigated as both an assessment tool and a rehabilitation strategy in PD. The purpose of this focused systematic review is to analyse the benefits and limitations of retro walking in relation to the gait parameters and balance control of PD patients. *Materials and Methods*: A structured literature search (2015–2025) was conducted across multiple databases in accordance with PRISMA (Preferred Reporting Items for Systematic Reviews and Meta-Analysis) guidelines. Eligibility criteria, screening procedures, and qualitative synthesis methods were predefined. Nine studies (including two randomized controlled trials) met the inclusion criteria. Methodological quality was assessed using PEDro and ROBINS-I tools, and the certainty of evidence was evaluated using GRADE. *Results*: The research results indicate within-group improvements in balance and gait parameters following BW training. Some of the included studies also suggest that BW may be a sensitive marker of balance deficits and fall risk. However, the evidence is limited by small sample sizes, heterogeneity of interventions, and a predominance of non-randomized designs. *Conclusions*: Current evidence regarding BW in PD remains preliminary. While BW may be considered as a supplementary component of rehabilitation, its specific efficacy cannot be clearly distinguished from general exercise effects. Further high-quality randomized controlled trials with standardized protocols and long-term follow-up are required.

## 1. Introduction

Parkinson’s disease (PD) is a progressive, age-associated neurodegenerative disorder, characterized by the degeneration of dopamine-producing neurons in the basal ganglia, a region of the brain critical for motor control [1]. PD presents with motor-related and nonmotor-related symptoms. Among nonmotor manifestations, cognitive deficits are relatively common and tend to progress more rapidly in PD, particularly in domains such as executive function, attention, and visuospatial abilities compared to cognitively healthy peers of similar age [2,3]. Gait and balance disturbances are frequently observed in PD and constitute significant motor impairments [4]. Gait disturbances include hypokinesia, reduced walking speed, diminished swing phase, increased variability in gait patterns, reduced stride length, and pronounced asymmetry in step timing [5,6]. Difficulties with gait and postural stability substantially affect patients’ quality of life by decreasing functional independence and increasing the risk of falls. Although pharmacological treatment is generally effective in alleviating cardinal motor symptoms of PD, gait and balance impairments tend to respond less favorably to dopaminergic therapies [7,8].

During activities of daily living (ADLs), individuals naturally engage in multidirectional walking, including lateral and backward movements, often without conscious awareness [9]. However, when performed deliberately, backward walking (BW) presents greater complexity, requiring higher levels of coordination. Research shows that BW speed declines with age and correlates with increased fall risk [10,11]. As a result, BW is frequently incorporated into physiotherapeutic interventions aimed at enhancing gait performance and lower limb mobility. Regular practice of BW has been shown to improve joint range of motion, muscular strength, and motor coordination in populations with neurological or musculoskeletal disorders, such as children with cerebral palsy [12,13]. This form of locomotion, although essential for daily activities such as turning, sitting back, or moving backward, poses a significant challenge for older adults [10,14]. In the context of PD, studies suggest that BW is more severely impaired than forward gait, highlighting its potential as a sensitive marker of motor dysfunction in this population [7,15,16].

In recent years, BW has gained increasing attention as a viable and innovative approach in rehabilitation aimed at enhancing gait and overall mobility [17,18]. Studies involving various neurological and developmental populations, such as individuals recovering from stroke and children with cerebral palsy, have shown that BW training, whether performed on a treadmill or over ground, can significantly improve multiple gait-related outcomes. These include increases in walking speed, stride and step length, improved gait cycle dynamics, reduced asymmetry, enhanced balance, and better functional capacity [17]. Notably, incorporating BW into conventional physiotherapy programs has also been associated with improvements in postural control [19,20].

Despite encouraging evidence supporting the effectiveness of BW training in various clinical populations, its application in individuals with PD remains insufficiently investigated. To date, no systematic review has comprehensively evaluated the clinical application of BW in individuals with PD. This gap is particularly important, considering that PD is frequently accompanied by backward gait disturbances, postural instability, and an increased risk of falls. Given these challenges, there is a clear need to critically synthesize the available scientific data regarding the use of BW in this population. Therefore, the objective of this review is to conduct a qualitative synthesis on the use of BW as a rehabilitation strategy in individuals with PD, with the aim of evaluating its clinical effectiveness and informing evidence-based therapeutic approaches.

## 2. Materials and Methods

This focused systematic review was based on the PRISMA guidelines [21,22]—A completed PRISMA checklist is included in the Supplementary Materials.

**Registration:** Due to the limited scope of this study and its classification as a focused systematic review, the review protocol was not prospectively registered in PROSPERO.

### 2.1. Eligibility Criteria

To guide this focused systematic review, a structured PICO framework was applied. The study population (P) included individuals with Parkinson’s disease at various stages of the disease, including patients with mild-to-moderate symptoms (H&Y I–II), patients in the early stages of de novo disease and patients with longer disease duration (5–10 years), as well as individuals with various clinical characteristics, such as cognitive function (MoCA < 17), autonomic symptoms (results of Scales for Outcomes in Parkinson’s Disease–Autonomic Dysfunction), incidence of falls, and the presence of freezing of gait (FOG). The intervention (I) comprised training using backward walking (BW). Various standardized clinical tests and instrumented tools were used to assess functional ability and gait parameters, including the 3-Meter Backward Walk Test (3MBWT), the 50-Foot Walk Test (50FWT), the Timed Up and Go Test (TUG), and the Five Times Sit to Stand Test (FTST). Gait analysis was performed using motion capture systems and computerized gait analysis. Some studies also investigated the impact of pharmacological interventions and gait training. The Comparison (C) involved contrasts between test conditions (e.g., forward walking vs. BW, OFF vs. ON medication), participant subgroups (e.g., fallers vs. non-fallers), and test repetitions to assess reliability, along with comparisons between PD participants and healthy controls. The Outcomes (O) focused on measures of gait and mobility performance, test–retest reliability, and the validity of clinical assessments. Additional outcomes included motor symptoms, cognitive and autonomic dysfunction, freezing of gait, and fall risk. This structured approach supports a comprehensive synthesis of evidence on BW as a potential tool to support rehabilitation strategies in patients with PD [8,16,19,23,24,25,26,27,28].

### 2.2. Search Strategy

The multi-search engine of the Main Library of the Medical University of Gdansk was used to search for publications. The review was based on research material obtained from 7 databases: Medline, Directory of Open Access Journals, Science Direct, Springer Journals, Journals Ovid, Research Gate, and *Journal of Clinical Neurology*. Study dates ranged from 2015 to 2025. An initial search was performed in May 2025 and repeated in July 2025 prior to the final review (the database was last searched on 28 July 2025). The search strategy was based on three categories of keywords: (1) terms related to the target population, (2) terms referring to walking direction, and (3) terms related to rehabilitation strategies. The Boolean operator “AND” was used to combine the keyword sets. Category (1) focused on individuals with PD and included terms such as “Parkinson’s disease”, “idiopathic Parkinson’s disease”, “Idiopathic Parkinson”, and “Parkinson”. Category (2) included terms associated with BW, such as “backward walking”, “backward gait”, “backward locomotion”, and “retro gait”. Category (3) addressed rehabilitation strategies and included terms such as “rehabilitation”, “rehabilitation strategy”, “physiotherapy”. These three categories of keywords were ultimately combined in the final search using the Boolean operator “AND” to intersect category (1) with category (2) and category (3). Final search query for each database was formulated as follows: (“Parkinson Disease” [Mesh] OR “Parkinson’s disease” OR “Idiopathic Parkinson*”) AND (“Backward Walking” OR “Backward Gait” OR “Retro Gait”) AND (“Rehabilitation” OR “Physiotherapy” OR “Rehabilitation Strategy”). This search string was applied across all selected databases, targeting the title, abstract, and keywords fields, to retrieve studies relevant to backward walking as a rehabilitation strategy in individuals with Parkinson’s disease.

### 2.3. Selection of Articles

The analysis included studies involving patients with idiopathic Parkinson’s disease, both in the early stages of the disease (de novo patients) and in the moderate stage, in which backward walking was assessed, in comparison to forward walking or other motor tasks, such as a 360° turn or dual-task challenges. Eligible studies included primary research, both randomized controlled trials (RCTs) and observational studies, that included clinical assessments and biomechanical gait parameters, including speed, stride length, stride variability, asymmetry, balance, and postural functions, published in peer-reviewed journals, in the period 2015–2025 (only the latest studies), and written in English. For a study to be eligible for analysis, it had to demonstrate a direct connection to the review’s topic by evaluating backward walking as a rehabilitation strategy for people with Parkinson’s disease. Additionally, the included publications had to present precisely defined results relevant to the objectives of the review.

Publications were excluded if the studies did not include patients with Parkinson’s disease, did not address backward walking, assessed only the usefulness of specific scales or rehabilitation devices (e.g., treadmills), did not present study results or were the following types of studies: case reports, case studies, abstracts, editorials, letters to the editor, reviews, and meta-analyses as well as grey literature.

It should be noted that the limited number of included studies reflects the emerging nature of the topic and the application of predefined eligibility criteria.

### 2.4. Aim and Objectives

This focused systematic review aims to critically evaluate the effectiveness of BW as a rehabilitation strategy in individuals diagnosed with PD. The primary objective is to assess its impact on gait performance, balance, and motor function based on quantitative and qualitative outcome measures. Furthermore, the review seeks to synthesize and interpret current evidence regarding the structure, implementation, and clinical outcomes of BW interventions within this population.

### 2.5. Data Extraction

Seventy-three records (Medline—11, Directory of Open Access Journals—3, Science Direct—28, Springer Journals—10, Journals Ovid—18, Research Gate—1, *Journal of Clinical Neurology*—2) were identified and narrowed to the topics mentioned in paragraph 2.2 Search Strategy. The initial screening of titles and abstracts was independently conducted by two reviewers to identify studies that met the inclusion criteria, and duplicate records were removed. Disagreements between reviewers were resolved through discussion. If consensus could not be reached, a third reviewer was consulted.

Twenty-two records were removed as duplicates; additionally, nine publications unrelated to the topics of “backward gait” and “Parkinson’s disease” were excluded, as were two articles for which the full text was not available. Forty articles of potentially eligible studies were then assessed in detail. As a result, 31 studies were excluded: 6 of them did not directly concern patients with PD, 21 did not analyse backward walking in groups of PD individuals, 2 focused on evaluating the utility of specific tools or scales in rehabilitation (e.g., treadmills), and another 2 were systematic reviews or meta-analyses. The remaining 9 publications were downloaded in full and assessed for compliance with the inclusion and exclusion criteria. None of the reports were rejected at this stage, and all 9 studies met the review criteria. As a result, the review conducted according to the PRISMA method yielded 9 papers (2 RCTs and 7 non-RCTs), which are shown in Table 1.

The following data were systematically extracted from each included study: (1) general study characteristics, including year of publication and study design; (2) participant information, such as sample size, mean age, and PD stage; (3) details of the BW intervention, including duration, frequency, and whether the intervention was supervised; (4) outcome measures related to gait performance, motor function, and balance; and (5) key results and conclusions relevant to the research question.

### 2.6. Synthesis Methods

Due to the heterogeneity of study designs, intervention protocols, and outcome measures, a quantitative meta-analysis was not feasible. Therefore, a qualitative narrative synthesis was performed. Studies were grouped according to study design and research objective (e.g., observational studies evaluating backward gait characteristics and randomized controlled trials evaluating rehabilitation interventions). Studies included in this review were evaluated for eligibility for synthesis by comparing their intervention characteristics, outcomes, and study designs against the predefined criteria described in Section 2.1. Eligibility Criteria. Data extracted from individual studies were organized into structured tables to allow clear presentation and comparison of key variables, including participant characteristics, intervention details, and outcome measures; no statistical conversions, imputations, or additional data manipulations were performed. A qualitative narrative synthesis was conducted to summarize and interpret the findings, with rationale based on the heterogeneity of study designs, small sample sizes, and variations in outcome measures, which precluded meta-analysis. Given the narrative synthesis approach, no sensitivity analyses were performed to assess the robustness of the results. This method allowed for a transparent overview of backward walking interventions and their reported effects on gait, balance, and motor outcomes in patients with Parkinson’s disease.

### 2.7. Quality Assessment and Risk of Bias

To improve methodological transparency, the quality of included studies was systematically assessed. Randomized controlled trials were evaluated using the PEDro scale, while non-randomized studies were assessed using the ROBINS-I tool. The overall certainty of evidence was further appraised using the GRADE framework.

## 3. Results

### 3.1. Study Selection

**Figure 1:**
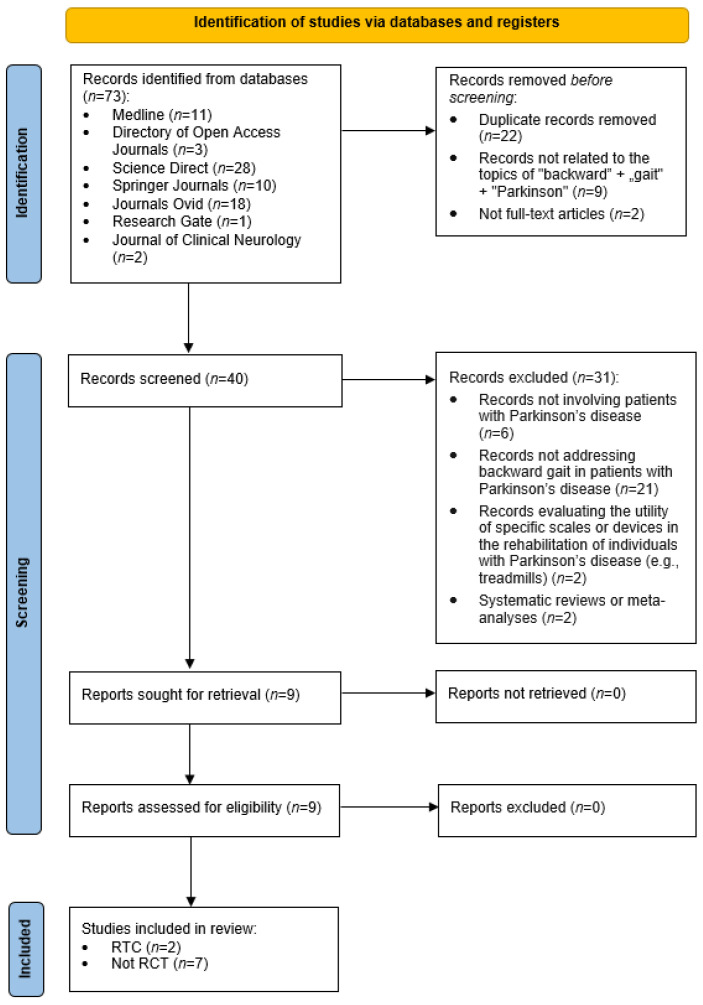
Flow chart adapted from PRISMA, which shows the process for identifying and screening the articles for inclusion and exclusion.

A total of 73 potentially relevant publications were identified through the search strategy. After excluding duplicate records (n = 22), those unrelated to the topic (n = 9), and records lacking full-text access (n = 2), 40 records remained for screening. Following the exclusion of studies that did not meet the inclusion criteria (n = 31), nine full-text articles were assessed. These comprised seven observational studies and two randomized controlled trials evaluating backward walking interventions in individuals with Parkinson’s disease. The review was conducted in accordance with the PRISMA statement [21,22] (Figure 1).

An analysis of the individual studies included in the review shows a high convergence in the inclusion and exclusion criteria for patients in the study group (Table 2). The average age of the participants was between 65 and 69 years old. Intervention sample sizes varied widely (e.g., in Tseng’s study n = 22, in Pongmala’s study n = 78).

The majority of studies on backward walking (BW) have primarily focused on evaluating functional gait parameters, balance, and motor coordination. Umar et al. (2024) [29] compared a BW training group with a standing balance training group, with the primary aim of assessing changes in the Berg Balance Scale and improvements in functional gait parameters. Grobbelaar et al. (2017) [19] compared BW and forward walking (FW) training, measuring outcomes including the MDS-UPDRS III, 10-Meter Walk Test (10mWT), and MoCA, with a focus on enhancements in gait, balance, and selected motor symptoms. Pongmala et al. (2025) [30] investigated the relationships between BW and forward gait in relation to balance function, motor symptoms, and body metrics, suggesting that BW may serve as an indicator of fall risk. Kellaher et al. (2022) [31] examined limb coordination during BW, while Son et al. (2022) [15] characterized gait disturbances associated with freezing of gait (FOG). Gilmore et al. (2019) [26] conducted a factor analysis of BW parameters in the context of overall gait function. Several observational studies, including those by Kwon et al. (2019, 2024) [8,16] and Lee et al. (2023) [27], not only assessed patients’ functional status but also suggested the potential of BW as a diagnostic or prognostic tool. Notably, a prospective study by Kwon et al. (2024) [8] demonstrated that BW parameters may predict the risk of future falls in patients with de novo Parkinson’s disease (PD).

Below is a summary of observational publications, outlining the therapeutic approaches applied, the assessment tools and tests employed, as well as the key findings.

#### 3.1.1. Differential Associations Between Retro Walking Versus Forward Walking, Axial Motor Features and Body Composition in Parkinson’s Disease

The study by Pongmala [30], published in 2025 in Parkinsonism and Related Disorders, investigated the differential associations between retro walking and forward walking performance, axial motor features, and body composition in PD. In this retrospective cross-sectional study, 78 individuals with idiopathic PD (54 males, 24 females; mean age 69.15 ± 5.61 years) underwent standardized assessments of retro and forward walking over an 8.5-m walkway, with walking times recorded under controlled laboratory conditions. Clinical evaluations included PIGD motor features (falls, impaired balance, freezing of gait), body composition measured by dual-energy X-ray absorptiometry (lean mass, fat mass, appendicular lean mass index, waist circumference), and balance performance assessed with Mini-BESTest sub-scores. Multivariate regression analyses revealed that retro walking time was significantly associated with impaired balance (OR = 1.111, df = 1, *p* = 0.028; χ^2^ = 17.727, *p* = 0.001) and showed a borderline association with a history of falls (OR = 1.168, df = 1, *p* = 0.027; χ^2^ = 9.236, *p* = 0.055). In contrast, forward walking time was specifically associated with freezing of gait (OR = 1.297, df = 1, *p* = 0.047; χ^2^ = 11.87, *p* = 0.018). In the multivariate linear regression model predicting retro walking time, the overall model was highly significant (F = 15.288, df = 8, *p* < 0.001). Retro walking time correlated with poorer reactive postural control (β = −3.964, *p* < 0.01), lower lean mass (β = −0.0003, *p* = 0.01), higher waist circumference (β = 1.304, *p* = 0.001), and lower appendicular lean mass index (β = −1.754, *p* = 0.016). The model predicting forward walking time was also significant (F = 5.474, df = 4, *p* < 0.001), with Mini-BESTest dynamic gait emerging as the only significant predictor (β = −0.775, *p* = 0.002). These findings suggest that retro walking may be particularly useful for assessing balance impairments and fall risk in PD, whereas forward walking is more relevant for evaluating freezing of gait.

#### 3.1.2. Persons with Parkinson’s Disease Show Impaired Interlimb Coordination During Backward Walking

The study by Grace K. Kellaher [31], published in Parkinsonism and Related Disorders in 2022, investigated interlimb coordination during backward versus forward walking in PD. Eighteen individuals with mild-to-moderate PD and 18 age- (±2 years) and gender-matched older adult controls were assessed using three-dimensional motion capture while walking forward and backward at their preferred speeds. Interlimb coordination was measured via the point estimate of relative phase, alongside spatiotemporal gait parameters and range of motion. Results showed that while forward walking coordination was comparable between groups, individuals with PD exhibited significantly impaired coordination during BW, particularly involving the more affected shoulder with the more affected hip and the more affected shoulder with the less affected hip, which was not explained by joint range of motion differences. This impairment occurred despite the absence of a significant Group × Condition interaction for basic gait parameters (λ = 0.810, F(5,30) = 1.41, *p* = 0.250, ηp^2^ = 0.19) and no group differences in joint ROM (λ = 0.788, F(4,31) = 2.09, *p* = 0.106). Importantly, BW itself produced large directional effects on gait—markedly slower speed (*p* < 0.001, ηp^2^ = 0.674), shorter step length (*p* < 0.001, ηp^2^ = 0.895), wider step width (*p* < 0.001, ηp^2^ = 0.607), and greater double support time (*p* < 0.001, ηp^2^ = 0.407) and significantly reduced ROM across all hip and shoulder joints (*p* < 0.001), confirming that the coordination deficit in PD was not attributable to reduced joint excursion but specifically to impaired inter-limb control during backward walking. The findings suggest that BW poses a unique motor coordination challenge for people with PD, highlighting the importance of tailored rehabilitation strategies to address these deficits.

#### 3.1.3. Turning Reveals the Characteristics of Gait Freezing Better than Walking Forward and Backward in Parkinson’s Disease

The study by Minji Son [15], published in Gait & Posture in 2022, aimed to determine whether turning movements reveal gait freezing characteristics more effectively than forward or backward walking in PD. A total of 68 PD patients diagnosed according to the UK Brain Bank criteria and 14 healthy older adults participated. For the forward and backward walking tests, 63 PD patients were included (28 with freezing of gait [FOG] and 35 without), while for the turning test, 57 PD patients participated (25 with FOG and 32 without). Participants completed forward walking, backward walking, and 360-degree turning tasks while gait parameters such as step length, walking speed, asymmetry index, and range of motion were assessed using a three-dimensional motion capture system. Results showed that turning, particularly 360-degree turns, magnified gait impairments in the FOG group compared to non-FOG patients and controls, with shorter step length, slower speed, higher asymmetry, and reduced range of motion, especially in the lower limbs. These deficits were less pronounced during straight walking. Consistent with this pattern, freezers demonstrated more severe motor impairment (UPDRS total: 49.1 ± 12.5; UPDRS III: 32.8 ± 9.9) and higher NFOGQ scores (11.9 ± 6.1) than non-freezers and controls. Correlation analyses further supported these findings: during forward walking, shorter step length (r = −0.289, *p* = 0.025), reduced walking speed (r = −0.268, *p* = 0.038), and lower toe-clearance height (r = −0.271, *p* = 0.036) were associated with worse performance. These associations strengthened during backward walking and especially during 360-degree turning, where step length (r = −0.359, *p* = 0.007), walking speed (r = −0.300, *p* = 0.022), toe-clearance height (r = −0.356, *p* = 0.007), and knee-joint ROM (r = −0.347, *p* = 0.009) showed significant correlations, accompanied by increases in total steps (r = 0.265, *p* = 0.049) and turning time (r = 0.299, *p* = 0.025). The findings indicate that turning is a more sensitive assessment tool than forward or backward walking for detecting and characterizing gait freezing in PD, with potential applications in clinical evaluation and targeted rehabilitation.

#### 3.1.4. Forward and Backward Walking in Parkinson’s Disease: A Factor Analysis

Gilmore [26] published in Gait & Posture an exploratory factor-analytic study evaluating whether BW provides added clinical insight beyond forward walking in PD. The cohort comprised 62 individuals with PD (tested both OFF and ON levodopa) and 11 age-matched healthy controls. Participants walked forward and backward at a self-selected pace on an instrumented walkway; spatiotemporal gait variables were extracted and submitted to factor analysis to identify underlying gait domains and to examine medication and direction effects. Across directions in the OFF state, four similar domains emerged—variability, rhythm, asymmetry, and pace—while in the ON state, a fifth domain related to posture appeared. In the OFF state, these four factors explained 70.4% of the variance in forward walking (with variability accounting for 30.0%, rhythm 14.5%, asymmetry 13.7%, and pace 12.2%) and 66.1% of the variance in backward walking (variability 17.8%, rhythm 17.0%, pace 17.0%, asymmetry 14.3%). Levodopa improved several forward walking parameters (notably speed, step length, and reduced variability/asymmetry), which was reflected in the ON-state factor structure, where five factors explained 72.8% of variance in forward walking and 75.6% in backward walking. In the ON state, posture emerged as an additional domain, though it accounted for a small proportion of variance (3.8% in forward walking and 7.7% in backward walking). Changes in gait with levodopa were smaller and less consistent during BW. The authors conclude that BW reflects the same core gait constructs yet is less responsive to dopaminergic therapy, suggesting complementary value for clinical assessment in PD.

#### 3.1.5. Backward Gait Is Associated with Motor Symptoms and Fear of Falling in Patients with De Novo Parkinson’s Disease

Kwon (2019) [16] published in the *Journal of Clinical Neurology* a study aimed at assessing the relationship between backward gait, motor symptoms, and fear of falling in patients with newly diagnosed PD. The study included 24 patients with de novo PD and 27 healthy control subjects. Participants performed three types of walking: forward gait, backward gait, and dual-task gait, with spatiotemporal parameters assessed using the GAITRite system. The results showed that patients with PD walked more slowly (*p* = 0.024), had shorter stride length (*p* < 0.001), and exhibited greater in-stride length variability (*p* = 0.044), particularly during BW. Backward gait speed was significantly correlated with bradykinesia score (rs = −0.469, *p* = 0.021), PIGD score (rs = −0.452, *p* = 0.027), and total motor score (rs = −0.473, *p* = 0.020) and the FFM score in patients with de novo PD was negatively correlated with the BG speed (r = −0.464 *p* = 0.022). The authors suggest that BW may be a sensitive indicator of early gait impairment and a potential biomarker for the progression of PD [16].

#### 3.1.6. Association Between Baseline Gait Parameters and Future Fall Risk in Patients with De Novo Parkinson’s Disease: Forward Versus Backward Gait

Kwon (2024) [8] published in the *Journal of Clinical Neurology* a prospective cohort study investigating the relationship between baseline gait parameters and the risk of future falls in patients with newly diagnosed, drug-naïve PD. Seventy-six patients meeting diagnostic criteria for de novo PD were recruited. Forward gait and backward gait were assessed using the GAITRite system, measuring spatiotemporal parameters such as gait speed, cadence, stride time, stride length, swing phase, double-support time, step width, gait variability (coefficient of variation for stride time and stride length), and gait asymmetry. Participants were followed for one year, with falls recorded during regular follow-up visits; 16 patients (21.1%) were classified as fallers. Compared with non-fallers, fallers demonstrated significantly slower gait speed (FG: *p* = 0.0184, BG: *p* = 0.0038) and shorter stride length in both forward and backward gait (FG: *p* = 0.0055, BG: *p* = *p*=0.0203), and greater stride-time variability specifically in backward gait (median [interquartile range): 7.72 [6.09–10.33] CV vs. 5.95 [4.72–7.45] CV, *p* = 0.0187). No significant group differences were observed for asymmetry parameters (FG: *p* = 0.6602, BG: *p* = 0.7646). Multivariable logistic regression revealed that slower baseline backward gait speed was an independent predictor of future falls, even after adjusting for age, sex, disease duration, cognitive score, and UPDRS-III motor severity, indicating that backward gait assessment may serve as a sensitive marker for early fall risk in PD.

#### 3.1.7. Association Between Gait and Dysautonomia in Patients with De Novo Parkinson’s Disease: Forward Gait Versus Backward Gait

Seon-Min Lee (2023) [27] published in the Journal of Movement Disorders a retrospective study examining the relationship between gait dynamics and autonomic dysfunction in patients with newly diagnosed, drug-naïve PD. The study included 38 patients, who were divided into two groups based on their scores in the Scales for Outcomes in Parkinson’s Disease–Autonomic Dysfunction (SCOPA-AUT): a high-score group (PD-HSAS) and a low-score group (PD-LSAS). Forward and backward gait were assessed using the GAITRite system, measuring parameters such as gait speed, cadence, stride length, stride time, swing phase, double-support phase, step width, and gait variability. Compared to the PD-LSAS group, the PD-HSAS group showed slower gait speed (*p* = 0.005), shorter stride (*p* = 0.050), decreased cadence (*p* = 0.008), increased double support phase (*p* = 0.003), decreased swing phase (*p* = 0.015), and greater variability in swing time (*p* = 0.018), with differences more pronounced in BW. Correlation analyses revealed that higher SCOPA-AUT scores were significantly associated with reduced gait speed (rs = −0.542, *p* = 0.002) in backward gait and stride length (r = −0.430, *p* = 0.012; rs = −0.429, *p* = 0.013) in forward gait and backward gait, the total SCOPAAUT score showed a significantly positive correlation with swing time variability (rs = 0.537, *p* = 0.001; rs = 0.463, *p* = 0.007), and greater double-support time (rs = 0.409, *p* = 0.018; rs = 0.479, *p* = 0.005), especially in backward gait. The authors conclude that autonomic dysfunction in early PD is closely linked to gait impairment, particularly during BW, and may serve as a predictor of early gait instability and fall risk.

A comprehensive comparison of the results of the individual studies mentioned above is presented in Table 3. A quantitative synthesis was not feasible due to high heterogeneity and small sample sizes.

Table 4 summarises publications based on randomised controlled trials (RCTs) and their characteristics according to the PRISMA methodology. Although the two studies applied different methodological approaches and measurement instruments—the Berg Balance Scale in one study [29] and the Instrumented 10-Meter Walk Test (i10mWT) with Mobility Lab (APDM, USA) in the other [19]—they both investigated the potential role of BW in patients with PD from slightly different perspectives. Despite these variations in outcome measures, the findings converged in demonstrating that incorporating BW exercises has a beneficial impact on functional performance in this patient population. The Friedman test showed significant within-group improvement (*p* < 0.001) in both groups from baseline to the 12th week in the Berg Balance Scale, with Wilcoxon pairwise comparisons indicating that each group improved significantly during the first month (*p* < 0.001), whereas no further significant change was observed in the second month (*p* ≥ 0.05). Similarly, findings from the study by Roné Grobbelaara, Ranel Ventera, and Karen Estelle Welmana showed that regular backward-walking training yielded meaningful within-group improvements in gait speed (*p* < 0.01), cadence (*p* < 0.01), step length (*p* = 0.02), and selected motor symptoms, including tremor (*p* = 0.02) and bradykinesia (*p* = 0.01), thereby confirming the beneficial impact of BW training on functional performance in individuals with Parkinson’s disease.

Taken together, these results suggest that BW may serve as a valuable therapeutic strategy to enhance both balance control and gait efficiency in individuals with PD, regardless of the specific assessment tools employed [19,29].

### 3.2. Risk-of-Bias in Studies

Table 5 presents an assessment of the risk of bias for seven observational studies (non-RCTs) using the updated version of the ROBINS-I tool (November 2025 version). Six domains were analysed: risk of bias due to confounding factors, intervention classification, participant selection, missing data, outcome measurement, and selection of reported outcomes. A low risk was assigned to the studies by Pongmala et al. (2025) and Kwon et al. (2024), meaning they were characterized by minimal risk of bias in each domain, taking into account the limited potential for uncontrolled confounding [8,30]. Moderate risk was identified in the studies by Kellaher et al. (2022), Son et al. (2022), Gilmore et al. (2019), Kwon et al. (2019), and Lee et al. (2023), mainly due to the potential influence of confounding factors, while the remaining domains were assessed as low risk [8,15,16,26,27,30,31].

Table 6 presents the assessment of the risk of bias in two randomized controlled trials (RCTs) according to the PEDro scale. Both Umar et al. (2024) and Grobbelaara et al. (2017) clearly defined participant eligibility criteria, used randomization with concealed allocation, and ensured comparability of groups at baseline [19,29]. Most data on key outcomes were obtained from over 85% of participants, and analyses were conducted according to the intention-to-treat principle. However, there was a lack of full blinding of participants, therapists, and outcome assessors, which is a common limitation in rehabilitation studies. Both studies scored 8 out of a possible 11 points, indicating good methodological quality with limitations primarily regarding blinding.

Despite the methodological limitations indicated, the results of the study suggest the potential effectiveness of rehabilitation strategies using backward walking in patients with Parkinson’s disease. However, it should be emphasized that the presence of systematic error in the studies limits the possibility of drawing clear conclusions and requires careful interpretation of the results. Further studies of higher methodological quality, using randomization, allocation concealment, and blinding of assessors, are necessary to confirm the effectiveness of this form of rehabilitation intervention.

### 3.3. Assessment of the Quality of Evidence

Based on the GRADE assessment of the non-RCTs [8,15,16,26,27,30,31], the overall quality of evidence supporting the effectiveness of backward gait (BG) in patients with Parkinson’s disease (PD) can be considered moderate or low (Table 7). Most studies were prospective observational studies, with one retrospective study [15], and included samples ranging from 24 to 78 participants. In these studies, the risk of systematic error was generally moderate due to small sample sizes, lack of complete randomization, and, in some cases, partial retrospective data collection. Nevertheless, the studies consistently reported improvement or correlations in BG parameters, suggesting high consistency of results. All studies directly measured backward walking using reliable and validated tools, such as 10-m walk tests with measuring devices or GAITRite systems, ensuring directness of evidence. Although precision was limited in smaller cohorts (n < 40), larger studies [8] provided more reliable estimates, alleviating concerns about inaccuracy. No signs of significant systematic error were found in the publications within the included studies. Importantly, several studies demonstrated factors that increased the certainty of the evidence, for example, showed clinically relevant associations between backward walking parameters and functional outcomes, including postural stability, fall risk prediction, and autonomic dysfunction [8,16,27]. In Gilmore’s study [26], pharmacological intervention (levodopa OFF vs. ON) further enhanced the observed improvement in BW, highlighting a dose-dependent effect. Although some studies were limited by small sample sizes and were not randomized, the moderate to high consistency of results, direct measurement of backward walking, and observed effect size provide moderate overall certainty regarding the clinical utility of backward walking measures in this population.

According to the GRADE assessment of the two RCTs [19,29] examining the effects of backward walking in individuals with Parkinson’s disease, the overall certainty of evidence is moderate (Table 8). Both studies were characterized by small sample sizes (n = 29–30), single-blind designs, and minor methodological limitations, which resulted in a downgrading for risk of bias. Despite these limitations, the consistency of findings remained high: both trials demonstrated improvements in clinically relevant gait and balance parameters following backward walking interventions, with no contradictory results between groups or outcomes. The studies directly measured outcomes related to balance and gait functionality in Parkinson’s disease, resulting in no downgrading for indirectness. Imprecision was present due to small sample sizes and, in Umar’s study [29], relatively wide confidence intervals around the primary effect estimate, leading to an additional one-level downgrade. There was no evidence of publication bias across the studies. Importantly, Umar’s study demonstrated a large and clinically meaningful effect of backward walking training on balance performance measured with the Berg Balance Scale, with a substantial mean difference and effect size sufficiently strong to merit a one-level upgrade for magnitude of effect. Study of Grobbelaar [19], while methodologically showing significant within-group improvements over time, did not reach the threshold for upgrading, as the effects were not of sufficiently large magnitude.

Although available studies suggest potential clinical benefits of backward walking in the rehabilitation of patients with Parkinson’s disease, the low quality of scientific evidence significantly limits the strength of the conclusions. The implementation of backward walking-based rehabilitation strategies in PD patients should be considered as a supplement rather than a primary intervention and should be used with caution. Limited certainty about the magnitude and durability of the effect, as well as the lack of data from large, well-designed RCTs, increases the risk of overestimating the effectiveness of this strategy. Therefore, the importance of further well-designed randomized controlled trials with larger samples, standardized intervention protocols, and safety assessments should be emphasized in order to clearly determine the effectiveness and validity of this rehabilitation strategy.

## 4. Discussion

This focused systematic review discusses the available evidence on the efficacy of BW as a rehabilitation strategy in PD. Available evidence provides preliminary indications that BW could influence motor and functional outcomes in PD, but the effects are inconsistent and its superiority over forward walking remains unconfirmed.

The review included two randomized controlled trials, both of which demonstrated improvements in the assessed parameters following the incorporation of BW into rehabilitation programs. The study on the effects of BW training and standing balance training on balance and fall risk among patients with PD suggested that BW might improve BBS scores to a slightly greater extent than standing balance training; however, both interventions yielded positive changes, and conclusions regarding BW-specific benefits should be drawn cautiously [29]. Similarly, the study by Grobbelaar, Venter, and Welman demonstrated that both forward and backward overground gait retraining effectively improved gait speed in patients with Parkinson’s disease; however, no significant differences were found between the two groups, which may represent a methodological limitation in distinguishing the specific benefits of backward gait training. Nevertheless, in line with these findings, multi-directional gait training showed improvements in cadence compared with a non-exercising control group [32]. While BW may contribute to these effects, the mechanisms—such as engagement of brainstem pathways—remain speculative and require further investigation. The intervention was further associated with an increase in step length, which is of particular relevance since individuals with PD typically exhibit shorter step length compared to age-matched healthy adults [19]. This gait characteristic is commonly attributed to bradykinesia, muscle rigidity, diminished muscular activation, and impaired kinaesthetic awareness, all of which compromise the efficiency and fluidity of locomotion [32]. Since people with PD often experience balance difficulties, it is likely that the BW group trained at only moderate intensities, as the task was unfamiliar and required an adaptation period. Therefore, BW should be introduced with caution and practiced in a safe setting with appropriate support. In general, current evidence suggests that BW yields positive outcomes in the rehabilitation of patients with PD, although it does not demonstrate substantial superiority over forward walking [19].

Gait speed in PD can be influenced by multiple factors, including motor symptoms. In the study Backward Gait is Associated with Motor Symptoms and Fear of Falling in Patients with De Novo Parkinson’s Disease, backward gait speed showed stronger associations with a broader range of motor impairments and was inversely correlated with the PIGD score in patients with de novo PD. However, as the study included only individuals with de novo Parkinson’s disease, it remains unclear whether these associations persist or change in later stages of the disease [16]. The results suggest that backward gait speed may serve as a useful surrogate marker of motor symptom progression or gait dysfunction. While the underlying mechanisms remain uncertain, it is plausible that backward gait, being less dependent on visual or cognitive input than forward or dual-task gait, is more directly linked to basal ganglia function. Consequently, backward gait may provide a more sensitive indicator of motor dysfunction, particularly in the early stages of PD [33,34,35].

Several factors contribute to fall risk in PD, including prior fall history, disease duration and severity, dyskinesia, freezing of gait, variability in stride time, postural instability, and fear of falling [36,37,38,39]. Falls may also occur early in the disease course, even in the de novo stage, although previous investigations into this issue have yielded inconsistent results [40,41,42]. In Backward Gait is Associated with Motor Symptoms and Fear of Falling in Patients with De Novo PD, the small number of participants with a recent fall limited meaningful comparisons between patients and controls. Nevertheless, fear of falling was found to be associated with backward gait speed, but not with forward or dual-task gait, in patients with de novo PD, whereas no such relationship was observed in healthy controls [16]. Consistent with this, Lord et al. identified slower gait as an independent predictor of falls in de novo PD [40], while Son et al. demonstrated that freezing of gait was more closely associated with decreased backward gait speed compared to forward gait speed [28]. Collectively, these findings suggest that backward gait speed may be more closely linked to fall risk than forward or dual-task gait in the early stages of PD, warranting further research into its potential as a predictor of falls [16].

Preliminary evidence suggests that gait control is not solely dependent on spinal pattern generators but may also rely substantially on cortical input, particularly for muscles essential to stability during touchdown [30]. It has been hypothesized that backward walking (BW), compared to forward walking (FW), engages the same cortical regions but to a greater extent in areas such as the motor and parietal cortices, thalamus, and basal ganglia, while possibly showing reduced activation of the cerebellum and brainstem [43]. These neural activation patterns may indicate that BW places higher demands on postural stability [44], which could explain its greater variability in stride time, stride length, and joint (hip and knee) range of motion, as well as reduced dynamic stability [14,44]. In the context of neurodegenerative diseases such as PD, it could be hypothesized that the increased cortical involvement required during BW reflects compensatory mechanisms for basal ganglia dysfunction and impaired cortical–subcortical integration [45]. Similarly, the observation that children with cortical deficits (e.g., cerebral palsy) exhibit impaired coordination during BW may support the notion that cortical and subcortical integrity is critical for this task. Taken together, BW might serve as a potential indicator of disrupted motor coordination and stability in disorders such as PD, where progressive degeneration of cortical, subcortical, and white matter networks is known to underlie gait dysfunction [46].

As demonstrated by previous studies, improvements in gait speed and dynamic balance are closely associated with a reduced incidence of falls [19], which is crucial for minimizing the risk of injuries. In PD, falls result from a combination of intrinsic factors, such as gait disturbances and freezing episodes, and extrinsic influences like environmental barriers. Effective recovery from slips or trips depends heavily on reactive postural control; however, this mechanism, particularly the ability to execute compensatory steps, is frequently impaired in both older adults and individuals with PD [47]. Evidence from the current analysis indicates that retro walking performance—especially prolonged completion time—was closely related to poorer reactive stepping responses and showed a near-significant association with fall occurrence. Although these findings may be partly explained by the small number of participants who provided reliable fall diary data, they nevertheless point to a possible link between retro walking duration and fall history. Because BW requires deliberate step execution and places greater demands on complex motor control, it may serve as an independent marker of fall risk in PD [30]. This interpretation is consistent with earlier research suggesting that retro walking is a more sensitive tool for distinguishing fallers from non-fallers in older populations [10]. Beyond its predictive potential, retro walking may have potential as a component of rehabilitation to target balance and fall risk [30,48]. BW has been shown to enhance gait speed, thereby contributing positively to the reduction of fall risk in patients with PD [29] through multiple mechanisms like engaging the neuromuscular system in an unconventional movement pattern, which promotes neuroplastic adaptations, which in turn support more effective sensory integration and motor coordination—both essential for maintaining balance [49,50]. However, evidence remains preliminary and does not conclusively demonstrate a clinically meaningful reduction in falls. Improvements in gait speed following BW have been reported, but the direct impact on fall risk is yet to be confirmed. In addition, BWT increases reliance on vestibular input and proprioceptive feedback, thereby strengthening postural control and improving sensory processing [49,51]. This type of training also activates muscle groups critical for gait, such as the hip extensors and ankle dorsiflexors, contributing to greater strength and refined movement control [19]. Furthermore, practicing BW requires continuous adjustments to dynamic stability and spatial orientation, especially when patients navigate obstacles or change direction [52]. Such tasks not only improve functional mobility but also build confidence, encouraging greater adherence to and participation in rehabilitation programs [29,53].

In addition, interesting research results were presented in the study by Tseng (2012) [54], which examined differences in forward and backward gait among early-stage, non-demented patients with Parkinson’s disease (PD) classified according to attention capabilities, compared to healthy controls. The authors found that gait impairments, such as slower walking speed, shorter stride length, and reduced swing phase, were more pronounced when walking backward. Notably, PD patients with poorer attention exhibited the most severe deficits, whereas those with better attention performed closer to the control group. These findings highlight the important role of attentional capacity in gait control, particularly for backward walking, and suggest that attention-enhancing strategies could be a valuable component of PD rehabilitation. However, as this study included only early-stage, non-demented PD participants, its findings may not generalize to individuals with more advanced disease, underscoring the need for future research encompassing later stages of Parkinson’s disease.

A critical limitation across the included interventional studies is the reliance on within-group analyses. In the absence of consistent and statistically significant between-group differences, it is not possible to attribute observed improvements specifically to backward walking. These effects may instead reflect general exercise benefits, learning effects, or increased familiarity with testing procedures.

In summary backward walking presents distinct biomechanical and neuromuscular characteristics compared to forward walking, including reduced walking speed, altered joint kinematics, reversed joint moments, and different muscle activation patterns. Despite being a less practiced and visually unsupported task, individuals demonstrate adaptive strategies—such as wider step width and shorter stride length—to maintain balance and stability. Overall, BW involves distinct motor control strategies and neuromuscular demands compared to FW, which could theoretically inform rehabilitation approaches, but evidence of its therapeutic advantage is still limited. However, further research involving broader populations is needed to generalize these results and fully understand the clinical relevance of backward gait training [55]. Considering that gait disorders are among the most common motor-related deficits in PD [56,57], it is crucial to expand the current body of research, particularly through well-designed randomized controlled trials, as evidence on BW in rehabilitation remains scarce due to the limited number of RCTs and methodological limitations in existing studies. Since BW has been shown to improve gait-related parameters [19], future investigations should focus on establishing its clinical relevance and determining its long-term effectiveness in this population. Moreover, it will be important to evaluate not only the role of BW as an independent intervention but also its potential when combined with other rehabilitation strategies to optimize therapeutic outcomes. Beyond gait and balance, future studies could examine potential effects of BW on cognitive functioning, quality of life, and neuroplasticity, although current evidence is insufficient to draw conclusions regarding these outcomes. Such investigations are needed to clarify whether BW has meaningful long-term therapeutic value.

## 5. Conclusions

This focused systematic review indicates that backward walking may have potential relevance in the assessment and rehabilitation of gait and balance impairments in Parkinson’s disease. However, the current evidence base is limited in both quantity and methodological quality. Although some studies report improvements following backward walking interventions, these findings are primarily based on small samples and within-group analyses, which limits causal interpretation. Therefore, backward walking should be considered a complementary rather than primary rehabilitation strategy. Future research should focus on well-designed randomized controlled trials with larger sample sizes, standardized intervention protocols, and long-term follow-up to determine the specific efficacy and clinical applicability of backward walking in this population.

In summary, until the results of larger controlled trials involving long-term follow-up are available, the actual efficacy of backward walking as a rehabilitation strategy in people with Parkinson’s disease cannot be clearly established.

### Limitations

Despite growing interest in backward walking in the rehabilitation of patients with Parkinson’s disease, several methodological and conceptual limitations must be acknowledged, which may constrain the interpretation and generalizability of the current findings.

First, the overall quality and quantity of the available evidence is limited. The focused systematic review included only two randomized controlled trials, each with relatively small sample sizes. For instance, Grobbelaar (2017) [19] demonstrated improvements in gait speed following both forward and backward overground gait retraining; however, no significant differences between the two groups were observed. Similarly, another RCT found that BW was more effective than static balance training in improving postural stability and reducing fall risk, though both interventions yielded positive outcomes (Umar et al., 2024) [29]. The small number of studies and participants reduces statistical power and makes it difficult to draw robust conclusions about the superiority or long-term effectiveness of BW.

Second, heterogeneity in intervention protocols across studies introduces additional complexity. Differences in training frequency, duration, intensity, and assessment tools make cross-study comparisons challenging. In some studies, BW was implemented alongside multi-directional or dual-task gait training, making it difficult to isolate its specific contribution to observed improvements [9,16,33,34,35,58].

Third, while some evidence suggests that BW may serve as a more sensitive marker of motor impairment and fall risk than forward walking, these associations remain inconsistent. For example, backward gait speed was found to inversely correlate with PIGD scores in patients with de novo PD (Kwon et al., 2019) [16], implying its relevance in assessing axial motor symptoms. However, the limited number of participants with a recent fall history restricted meaningful comparisons and weakened statistical reliability.

Moreover, the neurophysiological mechanisms underlying the benefits of BW remain insufficiently explored. Although some neuroimaging studies suggest that BW involves greater cortical activation—particularly in the motor and parietal cortices, basal ganglia, and thalamus—compared to FW, these findings are largely theoretical and lack clinical validation in PD populations (Godde et al., 2010) [43]. It is also unclear whether this increased cortical demand translates into functional gains or compensates for subcortical degeneration characteristic of PD.

Importantly, most studies were conducted in early-stage, non-demented PD participants, which restricts the generalizability of the findings to individuals with more advanced disease or cognitive impairment. For instance, Tseng, (2012) [54] demonstrated that gait impairments during backward walking (BW) were more pronounced in patients with attentional deficits, suggesting that cognitive factors may significantly influence BW performance. However, few studies have controlled for cognitive status or explored whether cognitive training interventions could enhance BW-related outcomes.

Finally, few studies included long-term follow-up assessments, making it uncertain whether the observed improvements in gait or balance persist beyond the intervention period. Additionally, the relationship between BW and broader outcomes such as quality of life, neuroplasticity, and cognitive functioning has not been sufficiently examined, despite their importance in PD rehabilitation.

These limitations highlight the urgent need for future high-quality, large-scale RCTs that utilize standardized protocols, include diverse patient populations, and incorporate long-term follow-up. Future research should also investigate the integration of BW with other therapeutic modalities and explore its effects on cognitive outcomes, neural adaptation, and overall quality of life in PD.

## Figures and Tables

**Table 1:** Description of articles initially included by PRISMA methodology.

Article Type	Focus	Reference
RTC with published results	Associations between backward walking training and standing balance training on balance and risk of fall	Muhammad Umar, Aamir Latif, Sameen Saeed, Shah Salman, Sharal Nayyer/2024 [29]
	Associations between backward and forward over-ground gait retraining	Ron’e Grobbelaara, Ranel Ventera, Karen Estelle Welman/2017 [19]
Other trials (not randomized and/or not controlled)	Associations between retro walking versus forward walking, axial motor features and body composition	Chatkaew Pongmala, Chernkhuan Stonsaovapak, Miriam van Emde Boas, Nicolaas Bohnen/2025 [30]
	Association between interlimb coordination and backward gait	Grace K. Kellaher, Sidney T. Baudendistel, Ryan T. Roemmich, Matthew J. Terza, and Chris J. Hass/2022 [31]
	Association between turning, forward gait, backward gait and gait freezing	Minji Son, Sang-Myung Cheon, Changhong Youm, and Jae Woo Kim/2022 [15]
	Forward and backward walking—a factor analysis	Greydon Gilmore, Arnaud Gouelle, Mitchell B. Adamson, Marcus Pieterman, Mandar Jog/2019 [26]
	Association between backward gait, motor symptoms and fear of falling	Kyum-Yil Kwon, Suyeon Park, Hye Mi Lee, Young-Min Park, Jinhee Kim, Jaehwan Kim, Seong-Beom Koh/2019 [16]
	Association between baseline gait parameters and future fall risk	Kyum-Yil Kwon, Jihwan You, Rae On Kim, Eun Ji Lee, Jungyeun Lee, Ilsoo Kim, Jinhee Kim, Seong-Beom Koh /2024 [8]
	Association between gait and dysautonomia	Seon-Min Lee, Mina Lee, Eun Ji Lee, Rae On Kim, Yongduk Kim, and Kyum-Yil Kwon /2023 [27]

**Table 2:** Inclusion and exclusion criteria for patients enrolled in the study programs.

Inclusion Criteria:
Age > 18 years A clinical diagnosis of idiopathic Parkinson’s disease Being at Hoehn and Yahr (H&Y) stages I and II Having no frank dementia (clinical dementia rating scale, CDR ≤ 0.5) Ability to transfer independently from sitting to standing, ability to locomotion Having adequate vision and hearing, with or without glasses and hearing aids, to read the study information sheet and hear the signals Ability to perform the forward and backward walking tests Acceptable history of falls
Exclusion Criteria:
Any primary neurodegenerative disorders other than PD Atypical clinical features suggestive of Parkinson plus syndrome or secondary parkinsonism during the follow-up period Individuals with serious musculoskeletal or medical problems Any comorbidities or complaints that could influence independent walking Inability to walk Significant cognitive impairment (MMSE score < 24) or major psychiatric disorder

**Table 3:** Comparative summary of the results of observational studies included in the review. Abbreviations: PG—Parkinson’s disease group; CG—Control group (healthy/no Parkinson’s disease group); FG—Forward gait; BG—Backward gait; PD-LSAS—low score of the Scales for Outcomes in Parkinson’s Disease-Autonomic Dysfunction (SCOPA-AUT) group; PD-HSAS—high score of the Scales for Outcomes in Parkinson’s Disease-Autonomic Dysfunction (SCOPA-AUT) group [8,15,16,26,27,30,31].

No.	Article	Parkinson’s Disease Group (PG)		Control Group (CG)	Unified Parkinson’s Disease Rating Scale (UPDRS) Part-III Total Score	PIGD (Postural Instability and Gait Disorder)	Hoehn and Yahr (HY) Stage	Walking Speed (cm/s)				Cadence (Steps/min)				Stride Length (cm)			
								Forward Gait (FG)		Backward Gait (BG)		FG		BG		FG		BG	
								PG	CG	PG	CG	PG	CG	PG	CG	PG	CG	PG	CG
1	Pongmala et al. (2025) [30]	78		x	47.73 ± 13.75	x	2.40 ± 0.51	x	x	x	x	x	x	x	x	x	x	x	x
2	Kellaher et al. (2022) [31]	18		15	35 ± 10	x	2.0 ± 0.5	92 (3.7)	108 (5.2)	63.7 (4.8)	79.3 (6.5)	96.77 (4.11)	108.05 (2.67)	100.79 (4.35)	107.57 (3.42)	56.6 (16)	57.4 (19)	36.6 (22)	40.8 (28)
3	Son et al. (2022) [15]	total	63	14	33.2 ± 7.9	x	2.5 ± 0.4	98 ± 25	113 ± 21	50 ± 18	71 ± 20	117.87 ± 11.98	115.65 ± 10.07	118.23 ± 17.90	110.76 ± 14.62	50 ± 10	60 ± 8	52 ± 18	77 ± 14
		freezer	28		32.8 ± 8.9	x	2.6 ± 0.4	91 ± 27		44 ± 18		115.03 ± 13.02		119.78 ± 18.65		47 ± 11		47 ± 20	
		non-freezer	35		33.6 ± 7.1	x	2.4 ± 0.4	104 ± 21		55 ± 17		120.14 ± 10.73		116.99 ± 17.45		52 ± 8		56 ± 15	
4	Gilmore et al. (2019) [26]	total	62	11	x	x	x	x	128.1 ± 17.1	x	100.4 ± 23.9	x	x	x	x	x	64.2 ± 5.9	x	48.1 ± 9.9
		ON levodopa	62		16.40 ± 8.21	x	x	120.4 ± 17.8		80.9 ± 21.1		x		x		61.1 ± 8.1		37.4 ± 9.3	
		OFF levodopa	62		29.68 ± 9.88	x	x	100.3 ± 23.4		63.0 ± 21.7		x		x		51.9 ± 11.5		29.9 ± 10.2	
5	Kwon et al. (2019) [16]	24		27	17.13 ± 6.40	1.54 ± 1.74	1.88 ± 0.40	107.72 ± 14.48	116.50 ± 9.92	66.76 ± 18.80	83.50 ± 13.54	114.35 ± 6.17	111.93 ± 9.08	111.31 ± 13.54	110.60 ± 11.19	113.35 ± 14.19	125.62 ± 8.75	72.56 ± 18.88	90.87 ± 11.09
6	Kwon et al. (2024) [8]	total	76	x	x	x	x	x	x	x	x	x	x	x	x	x	x	x	x
		non-fallers	60		24.08 ± 7.89	2 [1–2]	2 [2–2]	87.75 ± 20.13	x	64.41 ± 19.72	x	104.79 ± 9.44	x	113.04 ± 18.00	x	100.63 ± 18.58	x	69.56 ± 20.31	x
		fallers	16		22.06 ± 7.63	2 [1.75–3]	2 [2–2.12]	77.83 ± 9.61	x	50.37 ± 14.75	x	109.67 ± 10.85	x	107.51 ± 18.78	x	86.74 ± 15.71	x	57.44 ± 16.68	x
7	Lee et al. (2023) [27]	total	38	x	24.74 ± 11.77	2.53 ± 2.01	2.08 ± 0.39	x	x	x	x	x	x	x	x	x	x	x	x
		PD-LSAS	19		22.95 ± 10.64	1.74 ± 1.05	1.92 ± 0.34	51.80 ± 11.37	x	39.48 ± 11.52	x	102.31 ± 8.12	x	112.81 ± 16.21	x	60.96 ± 11.14	x	43.09 ± 12.43	x
		PD-HSAS	19		26.53 ± 12.85	3.32 ± 2.50	2.24 ± 0.39	45.10 ± 11.19	x	27.43 ± 11.84	x	101.38 ± 14.17	x	99.38 ± 14.89	x	53.89 ± 11.68	x	33.63 ± 12.17	x

**Table 4:** Summary of two randomized controlled trials comparing backward walking training to other interventions in Parkinson’s disease.

Authors/Year	Participants	Intervention	Outcomes Measurement	Results
Muhammad Umar, Aamir Latif, Sameen Saeed, Shah Salman, Sharal Nayyer /2024 [29]	N = 30 (17 men and 13 women) (n = 15 in the backward walking training group and n = 15 in the standing balance training group). Mean age for the participants was 50.03 ± 8.36 years. Parkinson’s patients at stage 3 or 4 (Brunnstrom motor recovery, lower extremity) able to walk 14 m with or without a walking aid or orthosis.	Both groups received TENS at 100 Hz frequency and 200 µs intensity, and a hot pack for 15 min each, along with ground walking training for 10 min, equal weight-bearing sit-to-stand exercises (10 repetitions, 2 sets), resistance exercises (leg press, leg extension, leg curl—10 repetitions, 2 sets each), and reaching tasks to improve balance. Group A underwent backward walking training (BWT) involving walking over the ground without assistive devices, 4 days per week for 12 weeks, 30 min per session, while Group B performed standing balance training (SBT), standing with eyes open on the right leg for 1 min and on the left leg for another minute, 4 days per week for 12 weeks, 30 min per session.	Berg Balance Scale	There was a significant within-group improvement (*p* < 0.001) in both groups from baseline to the 12th week in the Berg Balance Scale, with each group showing significant improvement (*p* < 0.001) in the first month, while no significant change (*p* ≥ 0.05) was observed in the second month; Group A, which received backward walking training (BWT), had a significantly larger mean difference in BBS score (22.13 ± 2.35 vs. 18.20 ± 3.58, 95% CI: 1.64–6.22) compared to Group B, which received standing balance training (SBT).
Roné Grobbelaara, Ranel Ventera, Karen Estelle Welman /2017 [19]	N = 29 (19 men and 10 women) (n = 14 in forward walking group (FWG) and n = 15 in backward walking group(BWG)) Two individuals were excluded during the course of the study, one (BWG) due to illness and one (FWG) due to injury (lost balance while turning around) that hampered them from completing the intervention. Mean age for the participants 71.0 ± 8.8 years. Participants had idiopathic PD with mild to moderate severity (H&Y stage II–III), could walk independently, had stable medication, no medical-attention injury in the past 3 months, no severe cognitive deficit (MoCA < 17), and no other conditions affecting locomotion, balance, or participation.	Participants completed three 45–60 min training sessions per week, with both the FWG and BWG following the same objectives but in opposite walking directions. The program progressively targeted posture and gait mechanics (pelvic tilts, abdominal activation, scapular setting, spinal lengthening, foot strike, push-off), gait tasks (weight shifts, step strategy, diagonal stepping, large strides), overground walking technique (marching, arm coordination, gait initiation and termination with cues), increasing walking speed, cadence, and distance (speed changes, continuous walking), directional changes (sideways walking, pattern walking, 90°/180°/360° turns), obstacle negotiation (stepping over objects, walking on narrow paths, manoeuvring through tight spaces), functional locomotion for daily activities (walking combined with tasks like bouncing a ball or folding cloth), and circuit training that integrated all previously learned skills.	Parkinson’s Disease Rating Scale (MDS-UPDRS) III, MoCA and Physical HealthQuestionnaire-9 (PHQ-9), Instrumented-10 m-walk-test (i10mWT) with Mobility Lab (APDM, Portland, OR, USA)	Both groups showed significant TIME effects for gait speed (usual and height-normalized, both *p* < 0.01), cadence (*p* < 0.01), and step length (*p* = 0.01), with within-group improvements in gait speed (FWG: +9.5%, *p* = 0.03; BWG: +14.0%, *p* < 0.01), cadence (BWG: +5.6%, *p* < 0.01), and step length (BWG: +6.9%, *p* = 0.02), but no between-group differences (*p* > 0.05). Tremor improved only in BWG by 12.7% (*p* = 0.02). Bradykinesia improved in both FWG (+23.6%, *p* < 0.01) and BWG (+12.2%, *p* = 0.01). Rigidity showed a TIME effect (*p* = 0.01) and a GROUP effect (*p* = 0.03), with worse scores post-testing in FWG (*p* = 0.05), contributing to a between-group difference (*p* = 0.04).

**Table 5:** Assessment of systematic error risk using the ROBINS-I—non-RCTs. Green color—low risk of bias, yellow color—maderate risk of bias.

Study	Domain 1: Confounding	Domain 2: Classification of Interventions	Domain 3: Selection of Participants into Study/Analysis	Domain 4: Missing Data	Domain 5: Measurement of Outcome	Domain 6: Selection of Reported Result	Overall Risk of Bias
Pongmala et al., 2025 [30]	🟩 Low (except uncontrolled confounding)	🟩 Low	🟩 Low	🟩 Low	🟩 Low	🟩 Low	🟩 Low
Kellaher et al., 2022 [31]	🟨 Moderate	🟩 Low	🟩 Low	🟩 Low	🟩 Low	🟩 Low	🟨 Moderate
Son et al., 2022 [15]	🟨 Moderate	🟩 Low	🟩 Low	🟩 Low	🟩 Low	🟩 Low	🟨 Moderate
Gilmore et al., 2019 [26]	🟨 Moderate	🟩 Low	🟩 Low	🟩 Low	🟩 Low	🟩 Low	🟨 Moderate
Kwon et al., 2019 [16]	🟨 Moderate	🟩 Low	🟩 Low	🟩 Low	🟩 Low	🟩 Low	🟨 Moderate
Kwon et al., 2024 [8]	🟩 Low (except uncontrolled confounding)	🟩 Low	🟩 Low	🟩 Low	🟩 Low	🟩 Low	🟩 Low
Lee et al., 2023 [27]	🟨 Moderate	🟩 Low	🟩 Low	🟩 Low	🟩 Low	🟩 Low	🟨 Moderate

**Table 6:** Assessment of systematic error risk using the PEDro scale—RCTs.

No.	PEDro Scale	Umar et al. (2024) [29]	Grobbelaara et al. (2017) [19]
1	Eligibility criteria were specified	yes	yes
2	Subjects were randomly allocated to groups (in a crossover study, subjects were randomly allocated an order in which treatments were received)	yes	yes
3	Allocation was concealed	yes	yes
4	The groups were similar at baseline regarding the most important prognostic indicators	yes	yes
5	There was blinding of all subjects	yes	yes
6	There was blinding of all therapists who administered the therapy	no	no
7	There was blinding of all assessors who measured at least one key outcome	no	no
8	Measures of at least one key outcome were obtained from more than 85% of the subjects initially allocated to groups	yes	yes
9	All subjects for whom outcome measures were available received the treatment or control condition as allocated or, where this was not the case, data for at least one key outcome was analysed by “intention to treat”	yes	yes
10	The results of between-group statistical comparisons are reported for at least one key outcome	yes	yes
11	The study provides both point measures and measures of variability for at least one	yes	yes
	Sum score:	8	8

**Table 7:** Assessment of the quality of evidence according to GRADE guidelines—not RCT. Orange color—low quality of the evidence, yellow color—maderate quality of the evidence, ↓ (down arrow) = lower the quality of the evidence, ↑ (up arrow) = raise the quality of the evidence.

Study	Risk of Bias	Inconsistency of Results	Indirectness of Evidence	Imprecision	Publication Bias	Factors Increasing Quality	Overall Quality
Pongmala et al. (2025) [30]	↓ 1 (partial randomization, small group)	↓ 1 (partially consistent FG/BG results)	0	↓ 1 (small sample)	0	0	🟠 Low
Kellaher et al. (2022) [31]	↓ 1 (observational, small group)	↓ 1 (different gait parameters FG/BG)	0	↓ 1 (small sample)	0	0	🟠 Low
Son et al. (2022) [15]	↓ 1 (retrospective, no randomization)	↓ 1 (partially consistent gait changes)	0	↓ 1 (medium sample)	0	0	🟠 Low
Gilmore et al. (2019) [26]	↓ 1 (interventional, non-randomized)	↓ 1 (some parameters consistent, some not)	0	0	0	↑ 1 (mild ON levodopa vs. OFF levodopa effect)	🟡 Moderate
Kwon et al. (2019) [16]	↓ 1 (small group, no randomization)	↓ 1 (variable results across gaits)	0	↓ 1 (small sample)	0	0	🟠 Low
Kwon et al. (2024) [8]	↓ 1 (observational, non-randomized, possible confounders)	↓ 1 (differences in FG/BG between fallers and non-fallers)	0	↓ 1 (few fall incidents)	0	↑ 1 (partial prognostic gait speed effect)	🟡 Moderate
Lee et al. (2023) [27]	↓ 1 (retrospective, non-randomized, patient selection)	↓ 1 (partially consistent SCOPA-AUT/gait associations)	0	↓ 1 (small sample)	0	↑ 1 (strong effect of autonomic-gait association)	🟡 Moderate

**Table 8:** Assessment of the quality of evidence according to GRADE guidelines—RCT. Yellow color—maderate quality of the evidence, ↓ (down arrow) = lower the quality of the evidence, ↑ (up arrow) = raise the quality of the evidence.

Study	Risk of Bias (Limitations)	Inconsistency of Results	Indirectness of Evidence	Imprecision	Publication Bias	Factors Increasing Quality	Overall Quality
Umar et al. (2024) [29]	↓ 1—single-blind only; baseline imbalance; non-probability sampling; small sample (n = 30)	0—direction of effect consistent; all outcomes improved as expected	0—direct measures of balance in PD	↓ 1—small sample; wide CI (95% CI: 1.64–6.22)	0—no evidence of selective reporting	↑ 1—clear, clinically meaningful superiority of BWT on BBS (large mean difference)	🟡 Moderate
Grobbelaara et al. (2017) [19]	↓ 1—single-blind, but allocation concealed; small sample (n = 29); 2 dropouts	0—both groups improve similarly; no contradictory findings	0—outcomes directly relate to gait function in PD	↓ 1—small sample; CI not reported but precision limited	0—no signs of reporting bias	0—effects significant over time but not large enough for upgrade	🟡 Moderate

## Data Availability

The data presented in this study are openly available in Medline, Directory of Open Access Journals, Science Direct, Springer Journals, Journals Ovid, Research Gate, and Journal of Clinical Neurology.

## Supplementary Materials

The following supporting information can be downloaded at: https://www.mdpi.com/article/10.3390/medicina62050867/s1, PRISMA 2020 Checklist.

## Abbreviations

The following abbreviations are used in this manuscript:

PD	Parkinson’s disease
BW	Backward walking
BG	Backward gait
FW	Forward walking
PHQ	Patient Health Questionnaire
H&Y	Hoehn and Yahr
CDR	Clinical dementia rating
MMSE	Mini-Mental State Examination
UPDRS	Unified Parkinson’s Disease Rating Scale
MDS	Movement Disorder Society
i10-min-WT	Instrumented-10 min-walking test
BWT	Backward walking training
FG	Forward gait
BWG	Backward walking group
FWG	Forward walking group
SBT	Standing balance training
MoCA	Montreal Cognitive Assessment
ADLs	Activities of daily living
SCOPA-AUT	Scales for Outcomes in Parkinson’s Disease–Autonomic Dysfunction
ROM	Range of motion
BBS	Berg Balance Scale
RCT	Randomized control trial
3MBWT	3-Meter Backward Walk Test
50FWT	50-Foot Walk Test
TUG	Timed Up and Go
FTST	Five Times Sit to Stand Test
PIGD	Postural Instability and Gait Difficulty
FOG	Freezing of gait
